# Superstructures with cyclodextrins: Chemistry and applications

**DOI:** 10.3762/bjoc.8.148

**Published:** 2012-08-16

**Authors:** Helmut Ritter

**Affiliations:** 1 Institute of Organic Chemistry and Macromolecular Chemistry II, Heinrich-Heine-University of Düsseldorf, Universitätsstraße 1, D-40225 Düsseldorf, Germany

**Keywords:** cyclodextrins, superstructures

Cyclodextrins (CDs) are cyclic oligosaccharides consisting of 6 (α), 7 (β) and 8 (γ) 1,4-glycosidically linked α-D-glucose units. Production of these compounds is accomplished by enzymatic degradation of starch using CD-glycosyl transferase.

Since their discovery more than 100 years ago, a significant development in CD research has taken place. Right after their discovery, CDs were very expensive due to the fact that only small quantities were available. In addition to this, it was assumed that CDs are toxic to human beings. Meanwhile, things have changed. Now, large quantities of CDs are available at low prices. Additionally, it could be demonstrated that CDs are not toxic to humans. Various applications of CDs can be found, e.g. the binding of odors and flavors. Even drugs can be complexed and set free after a while by CDs. This enables the transfer of hydrophobic substances into aqueous media, which is of great interest in pharmaceutical chemistry. For instance, release and molecular dispersion of antihistamines in the tear fluid are achieved very quickly by means of amylase catalysis.

Regarding the interactions of CDs with low-molecular and macromolecular compounds, respectively, the reactivity of complexed molecules is another key factor besides solubility effects. For example, differences in the absorption spectra of some compounds are caused by complexation with CDs.

For analytical purposes, CDs are used as chiral hosts. Thus, the application of CDs in column chromatography and HPLC to isolate and separate suitable racemic mixtures is broadly established. More recently, the use of CDs as food additives was approved. For example, dispersion of triglycerides can be achieved by the addition of α-CD.

In organic synthesis and polymer chemistry, the interest in CDs has increased significantly during the past decades. For instance, in the latter case covalent attachment of CDs to macromolecules and threading of CD molecules onto suitable macromolecular chains could be demonstrated. Unlike other macrocycles such as crown ethers or calixarenes, the cavity of CDs is big enough to include more or less hydrophobic molecules completely. In contrast to macrocyclic cucurbiturils, CDs show a better solubility in water. On top of that, they are also biodegradable.

Considering the importance of CDs as demonstrated above, we felt encouraged to summarize some of the latest results achieved in CD research. Contributions from leading research groups dealing with CDs can be found in this Thematic Series of the Beilstein Journal of Organic Chemistry.

Helmut Ritter

Düsseldorf, July 2012

